# Volumetric growth of the lungs in human fetuses: an anatomical, hydrostatic and statistical study

**DOI:** 10.1007/s00276-014-1269-7

**Published:** 2014-02-18

**Authors:** Michał Szpinda, Waldemar Siedlaczek, Anna Szpinda, Alina Woźniak, Celestyna Mila-Kierzenkowska, Marcin Wiśniewski

**Affiliations:** 1 Department of Normal Anatomy, The Ludwik Rydygier Collegium Medicum in Bydgoszcz, Łukasiewicza 1 Street, 85-821 Bydgoszcz, Poland; 2 Department of Medical Biology, The Nicolaus Copernicus University in Toruń, Karłowicza 24 Street, 85-092 Bydgoszcz, Poland

**Keywords:** Fetal pulmonary volume, Three-dimensional ultrasound, Magnetic resonance imaging, Water replacement, Regression analysis, Human fetuses

## Abstract

**Purpose:**

The prenatal assessment of lung volume is becoming increasingly important in determining survival in both preterm infants and newborns affected by pulmonary hypoplasia. This study aimed to examine the lung volumes in the human fetus at varying gestational ages.

**Materials and methods:**

Using anatomical, hydrostatic (water displacement according to Archimedes’ patent) and statistical methods (one-way ANOVA test for paired data and post-hoc Bonferroni test, Kolmogorov–Smirnov test, Levene’s test, Student’s *t* test, regression analysis), volumes of the right and left lungs were measured in 67 human fetuses of both sexes (35 males, 32 females) aged 16–25 weeks, derived from spontaneous abortions and stillbirths.

**Results:**

No male–female differences concerning the right and left pulmonary volumes were found. The mean volume of the right lung increased from 1.43 ± 0.25 to 8.45 ± 2.66 cm^3^, according to the cubic function *y* = –1.592 + 0.0007 × age^3^ ± 0.851 (*R* ^2^ = 0.84). The volumetric growth of the left lung, from 1.24 ± 0.22 to 6.78 ± 3.03 cm^3^, followed the cubic model *y* = –1.110 + 0.0005 × age^3^ ± 0.794 (*R* ^2^ = 0.78). The total pulmonary volume increased from 2.67 ± 0.47 to 15.22 ± 5.58 cm^3^, in accordance with the cubic model *y* = –2.729 + 0.0012 × age^3^ ± 1.598 (*R* ^2^ = 0.83). The mean volumes of the right and left lungs accounted for 54.9 ± 2.0 and 45.1 ± 2.0 %, respectively, of the total lung volume.

**Conclusions:**

No sex differences are found between the lung volumes in the fetus. The growth of fetal lung volume follows a three-degree polynomial function. Throughout the analyzed period the two lungs grow proportionately to each other, with the volumetric predominance of the right lung. The lung volumes in the fetus are of great relevance in the evaluation of the normal pulmonary growth and the diagnosis of pulmonary hypoplasia.

## Introduction

The prenatal assessment of lung volume is indispensable in determining survival in both preterm infants and newborns affected by pulmonary hypoplasia [1], due to renal malformations, oligohydramnios, fetal hydrops, skeletal dysplasias, congenital diaphragmatic hernia, intrathoracic masses, pulmonary malformations (congenital adenomatoid malformation, bronchopulmonary sequestration), and fetal akinesia [2–9]. The only two methods that enable clinicians to reliably measure fetal lung volume are three-dimensional ultrasound and MRI. Three-dimensional ultrasound allows determination of lung volume by stepping through the fetal thorax slice by slice, and then by building the sum of each slice’s volume. Lee et al. [10] indirectly obtained the total fetal pulmonary volume by subtracting the fetal heart volume from the internal thoracic volume. As a consequence, the resulting lung volume included great vessels, trachea, esophagus and thymus. Pöhls and Rempen [11] estimated separately each pulmonary volume with the use of conventional multiplanar imaging by building the sum of all the particular volumes of parallel slices. An improved three-dimensional ultrasound method constitutes the rotational technique with virtual organ computer-aided analysis (VOCAL) [9, 12]. This method allows lung volume calculation by rotating the organ around a fixed axis through a number of sequential steps [12]. Fetal lung volume determination by MRI is limited due to high cost, relatively long acquisition times, severe artifacts impaired by fetal movements, and low acceptance in pregnant women [10, 11].

To the best of our knowledge, no nomograms have been computed using detailed direct measurements of pulmonary volume in human fetuses. So as to supplement such information, in this study we aimed to concentrate on:

- age-specific references for volume of the right and left lungs at varying gestational ages,
- volumetric growth of the two lungs over time (growth curves), and
- the relative volumetric growth of the right and left lungs.

## Materials and methods

This examination was performed on 67 autopsied human fetuses of both sexes (35 males, 32 females) of white racial origin, which had been derived from spontaneous abortions or stillbirths during the years 1989–1999, as a result of placental insufficiency. As a prerequisite, the sample was built by rejection of fetuses from diabetic mothers or multiple pregnancies, and fetuses affected by congenital and chromosomal anomalies or intrauterine growth restriction. Legal and ethical considerations were approved by the University Research Ethics Committee (KB 190/2011). The gestational age ranged from 16 to 25 weeks (Table 1). The fetal ages in weeks were precisely assessed from the fetal crown-rump length (gestational age) [13], known date of the beginning of the last maternal menstrual period (amenorrhea age), and fine-tuned by a combination of abdominal circumference, femur length, and bi-parietal diameter known by early second-trimester ultrasound scan (ultrasound age).

**Table 1:** Age, number and sex of fetuses studied

Gestational age (weeks)	Crown-rump length (mm)				*n*	Sex	
	Mean	SD	Min	Max		Males	Females
16	111.0	4.2	108.0	114.0	2	1	1
17	122.1	3.7	115.0	126.0	8	4	4
18	136.7	4.3	130.0	142.0	10	5	5
19	153.3	2.0	150.0	155.0	6	4	2
20	161.6	3.3	156.0	166.0	14	7	7
21	174.4	3.8	170.0	180.0	7	2	5
22	188.2	2.5	185.0	190.0	5	3	2
23	195.8	1.8	193.0	198.0	6	4	2
24	208.3	2.9	205.0	212.0	6	4	2
25	220.0	0.0	220.0	220.0	3	1	2
Total					67	35	32

**Fig. 1:**
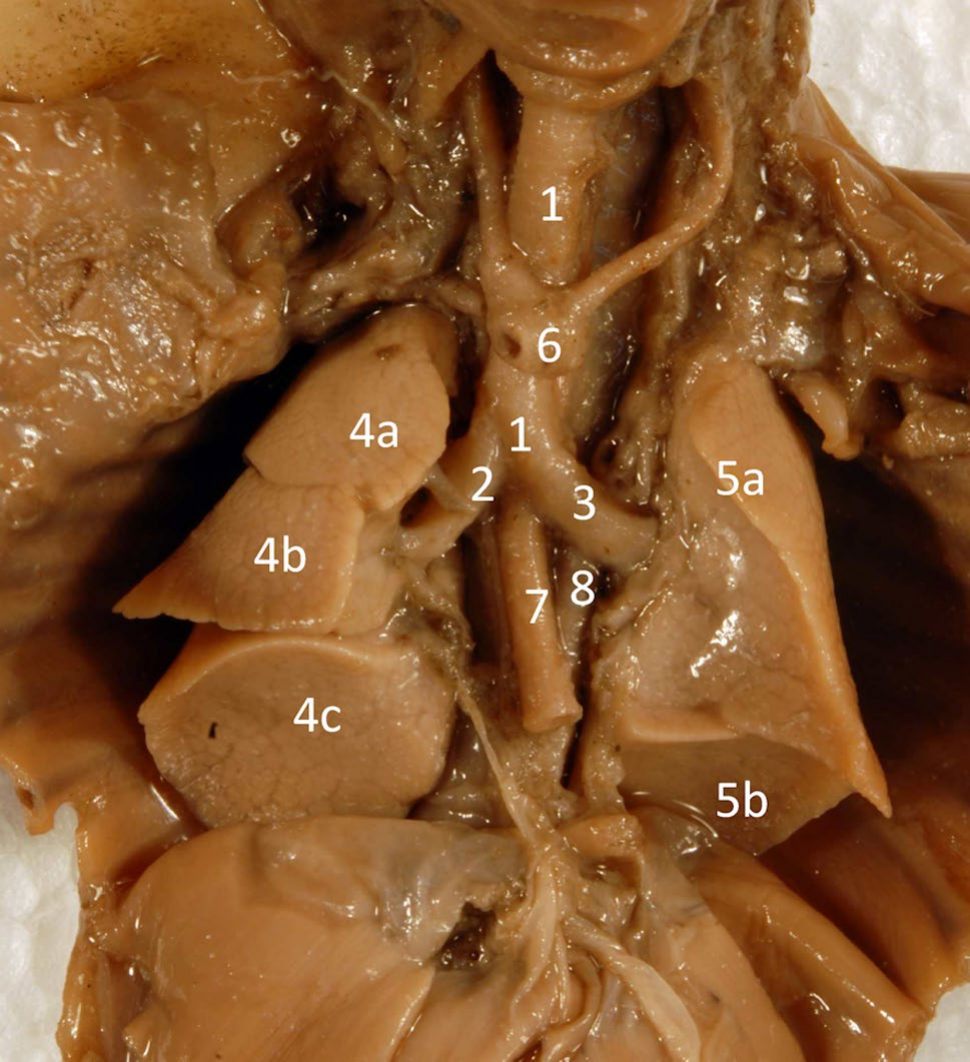
Thoracic viscera in situ (after removing the heart out) in a male fetus aged 19 weeks: *1* trachea, *2* right main bronchus, *3* left main bronchus, *4a* superior lobe of right lung, *4b* middle lobe of right lung, *4c* inferior lobe of right lung, *5a* superior lobe of left lung, *5b* inferior lobe of left lung, *6* aortic arch, *7* esophagus, *8* thoracic aorta

After having been immersed in 10 % neutral buffered formalin solution for 12–24 months for preservation, every fetus was dissected through sternotomy under tenfold magnification with the use of a stereoscope (Fig. 1). After opening the thorax, the root of each lung was dissected and cut off at the hilum, and then both lungs were removed out of the thorax. Neither external nor internal pulmonary malformations were macroscopically observed in every fetus, thereby the sample could be considered normal.

In fact, the 12- to 24-month formalin preservation certainly modifies the weight and density of fixed organs. As reported by Guihard-Costa et al. [14], the weight gain of the formalin-fixed organs may vary from 10 to 12 % for the brain to 20–25 % for the heart and liver, when compared to their initial weight. Furthermore, formalin fixation is responsible for volume modification of organs in question due to their tissue shrinkage. Of note, this is considerably expressed with relation to isolated organs [15]. On the contrary, formalin fixation has little influenced both shape and volume of the lungs in the material under examination. This results from the fact that completely degassed fetal lungs have been preserved in situ in the sealed thoracic cavity, fitting it perfectly. As it turned out, lung shrinkage was here minimized and comparable to thoracic-wall shrinkage at the level of 0.5–1.0 % [16].

**Fig. 2:**
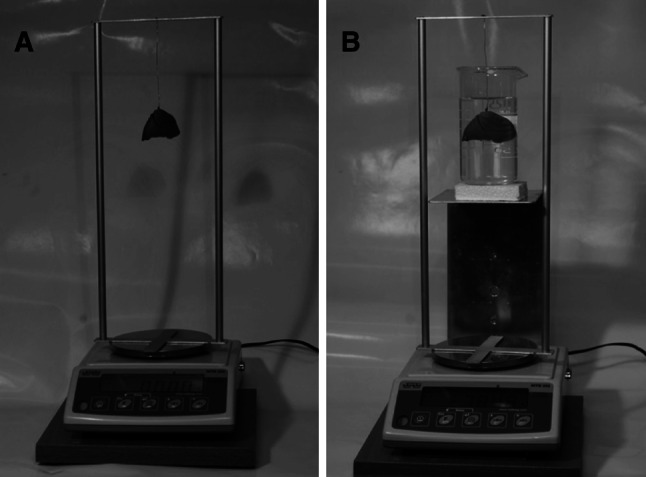
A double weighing procedure to obtain the weight of the lung in air (**a**) and distillate water (**b**)

Afterward, the absolute volume of every lung, as an object of complex shape, was measured using a hydrostatic method, based on Archimedes’ patent [17]. Therefore, a body submerged in water will lose weight quantitatively equal to the weight of the water displaced by the body. This required a double weighing procedure (Fig. 2) to obtain the weight (in g) of the lung in air (*W*_A_) and the weight (in g) of the lung in distillate water (*W*_W_) with the known specific gravity (in g/cm^3^) of water (*G*_W_) and air (*G*_A_) in the range of temperature between 14 and 20 °C, as displayed in Table 2. Thus, every measurement of the weight of the lung in air and water was accompanied by simultaneous measurements of the temperature of air and water.

**Table 2:** Specific gravity of water and air in the range of temperature between 14 and 20 °C [16]

Temperature (°C)	Specific gravity of water (g/cm^3^)	Specific gravity of air (g/cm^3^)
14.0	0.99927	0.00123
14.5	0.99920	0.00123
15.0	0.99912	0.00123
15.5	0.99904	0.00122
16.0	0.99895	0.00122
16.5	0.99887	0.00122
17.0	0.99878	0.00122
17.5	0.99869	0.00122
18.0	0.99860	0.00121
18.5	0.99850	0.00121
19.0	0.99840	0.00121
19.5	0.99831	0.00121
20.0	0.99820	0.00121

As a result, the volume of a lung (*V*) can be determined by the following formula:

$$V = W_{\text{A}} {-} \, W_{\text{W}} /G_{\text{W}} {-} \, G_{\text{A}}$$

It is noteworthy that the nominator *W*_A_ − *W*_W_ is completely independent of the weight gain of formalin-fixed viscera.

For every fetus the following four measurements and calculations of the lungs were performed:

1. volume in mm^3^ of the right lung,
2. volume in mm^3^ of the left lung,
3. total volume in mm^3^ of the right and left lungs, and
4. the left-to-right lung volume ratio.

In a continuous attempt to minimize measurement and observer bias, all measurements were carried out by one researcher (W.S.). Each measurement was performed three times under the same conditions but at different times, and then averaged. The differences between the repeated measurements, as the intra-observer variation, were assessed by the one-way ANOVA test for paired data. The data obtained were tested for normality of distribution (the Kolmogorov–Smirnov test) and homogeneity of variance (Levene’s test). As the first step in the statistical analysis, Student’s *t* test was used to examine how sex influenced the values obtained. So as to examine sex differences, first we checked differences between the following three age groups: 16–18 (*n* = 20), 19–21 (*n* = 27), and 22–25 (*n* = 20) weeks, and second for the whole sample, without taking into account fetal ages. To check whether or not significant differences existed with age, the one-way ANOVA test for unpaired data, and then post-hoc Bonferroni comparisons were used. Linear and nonlinear regression analysis was used to derive the best-fit curve for each volume studied against gestational age, with estimating coefficients of determination (*R* ^2^) between each parameter and fetal age. The relative growth of the two lungs was expressed as the left-to-right lung volume ratio. Differences were considered significant at *P* < 0.05.

## Results

**Fig. 3:**
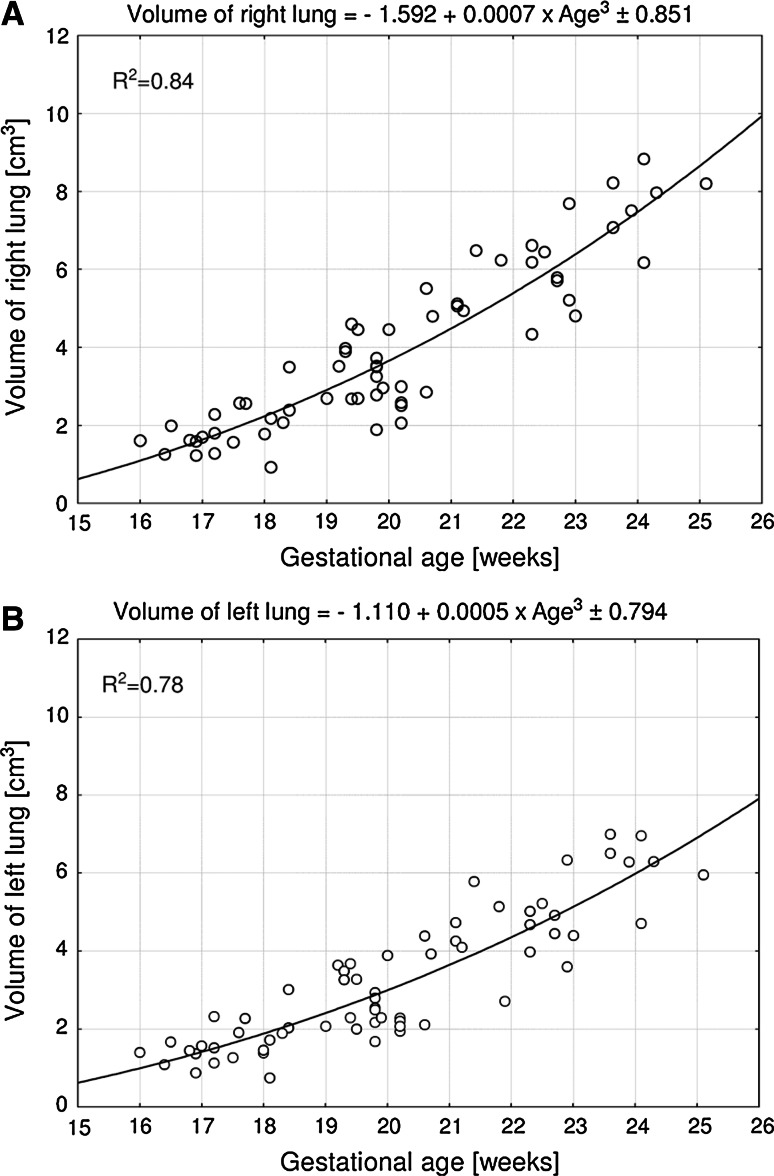
Right (**a**) and left (**b**) lung volumes versus fetal age

**Fig. 4:**
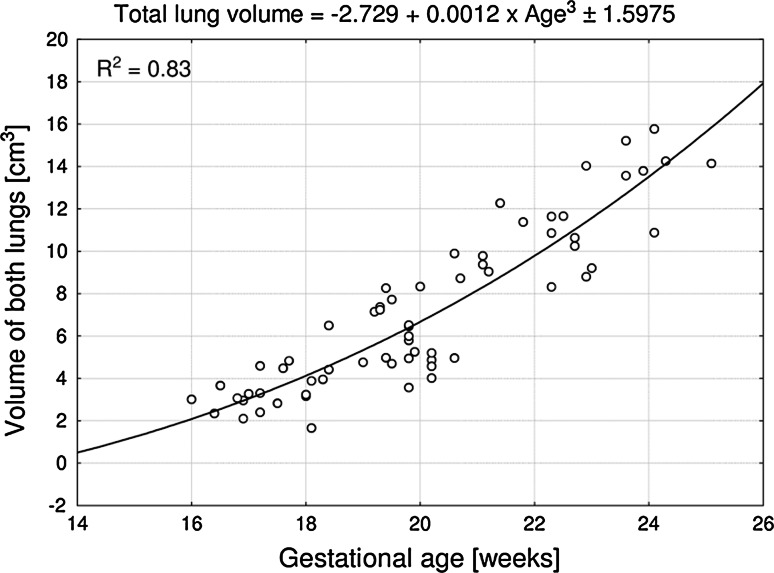
Total lung volume versus fetal age

No statistically significant differences were found in assessing the intra-observer reproducibility of lung volume measurements (*P* > 0.05, the one-way ANOVA test for paired data and post-hoc RIR Tukey test). Since no significant difference was observed in the values of lung volume according to sex (*P* > 0.05, Student’s *t* test), pulmonary volumetric data have been summarized for both sexes in Table 3. On the contrary, a statistically significant (*P* < 0.001, the one-way ANOVA test for unpaired data and post-hoc RIR Tukey test) increase in volumes of the lungs was found with advancing gestational age. Therefore, the relationships between pulmonary volumes and gestational age have been smoothed by fitting the mathematical models, as presented in Figs. 3 and 4. The best fits for pulmonary volumes with gestational age in weeks (*P* < 0.001) turned out to be three-degree polynomial functions.

**Table 3:** Fetal pulmonary volumes

Gestational age (weeks)	*n*	Volume (cm^3^)					
		Right lung		Left lung		Right and left lungs	
		Mean	SD	Mean	SD	Mean	SD
16	2	1.43^A^	0.25	1.24^B^	0.22	2.67	0.47
17	8	1.68^A^	0.35	1.49^B^	0.42	3.17	0.76
18	10	2.13^A^	0.69	1.77^B^	0.62	3.90	1.31

		↓(*P* < 0.001)		↓(*P* < 0.001)		↓(*P* < 0.001)	
19	6	3.10^A^	0.76	2.47^B^	0.71	6.63	1.42
20	14	3.56^A^	0.78	3.07^B^	0.59	5.56	1.35
21	7	4.96^A^	1.09	4.18^B^	1.10	9.15	2.18
22	5	5.27^A^	1.56	4.30^B^	1.00	9.57	2.54
23	6	5.94^A^	1.02	4.82^B^	0.92	10.76	1.90
24	6	7.63^A^	0.93	6.29^B^	0.83	13.92	1.71
25	3	8.45^A^	2.66	6.78^B^	3.03	15.22	5.58

Means in rows, marked by different letters A and B differ significantly (*P* < 0.001)

The right pulmonary volume was found to be greater than the left one (*P* < 0.001). The mean volume of the right lung increased from 1.43 ± 0.25 cm^3^ in the 16 weeks to 8.45 ± 2.66 cm^3^ in the 25 weeks of gestation. Scatterplots showing right lung volume against gestational age (Fig. 3a) followed the three-degree polynomial (cubic) function *y* = –1.592 + 0.0007 × age^3^ ± 0.851 (*R* ^2^ = 0.84). According to this model, the right lung volume grew by 0.54 and 1.19 cm^3^ for the 16 and 24 weeks, respectively. Between 16 and 25 weeks of gestation, the volumetric growth of the left lung from 1.24 ± 0.22 to 6.78 ± 3.03 cm^3^ followed according to the cubic model *y* = –1.110 + 0.0005 × age^3^ ± 0.794 (*R* ^2^ = 0.78) (Fig. 3b). This means that the left lung revealed an increase in volume by 0.41 cm^3^ in the 16 weeks and by 0.90 cm^3^ in the 24 weeks.

In the analyzed period, the total volume of the right and left lungs increased from 2.67 ± 0.47 to 15.22 ± 5.58 cm^3^. The volumetric growth of the two lungs changed as a function of gestational age, according to the three-degree polynomial formula *y* = –2.729 + 0.0012 × age^3^ ± 1.598 (*R* ^2^ = 0.83) (Fig. 4). As a result, the total lung volume revealed an increase in values by 0.98 cm^3^ in the 16 weeks and by 2.16 cm^3^ in the 24 weeks of gestation.

**Fig. 5:**
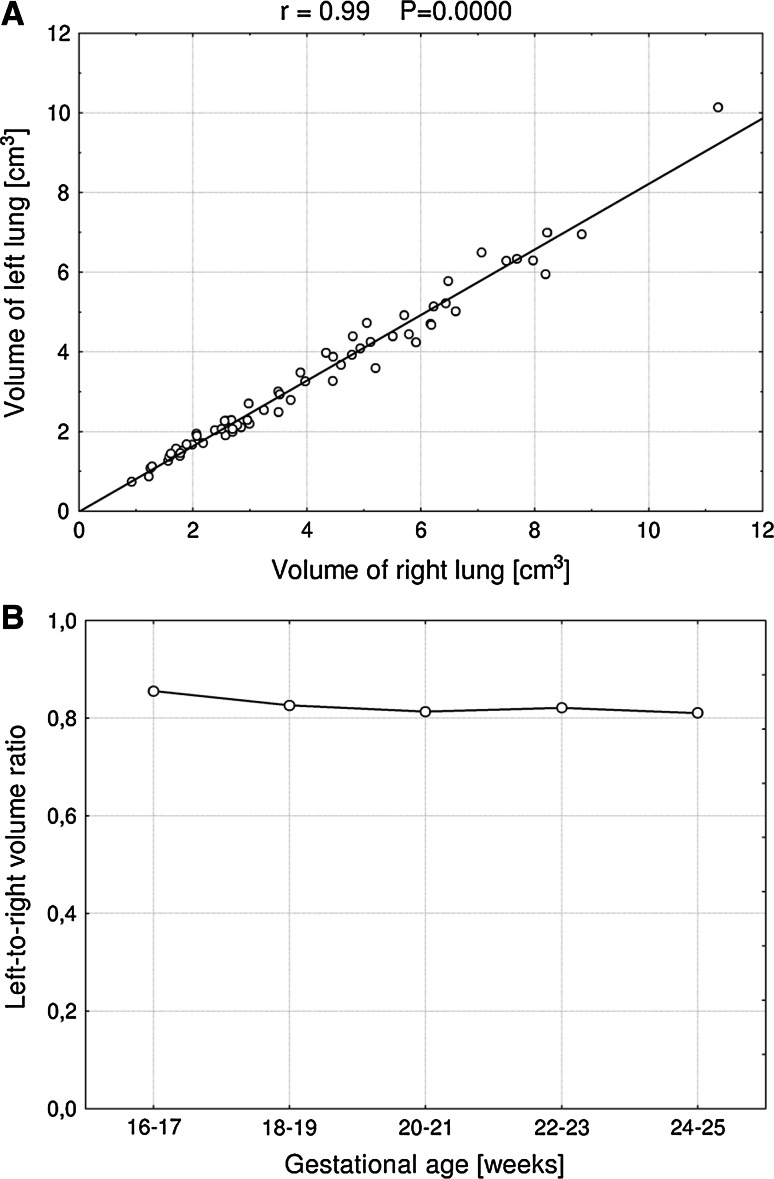
Proportionate growth of the left and right lungs

The rate of growth of the left and right lungs was alike (*r* = 0.99, *P* = 0.0000), because the left lung volume plotted against the right lung volume turned out to be a straight relationship (Fig. 5a). Furthermore, the left-to-right lung volume ratio was stable throughout the analyzed period (Fig. 5b). The mean volume of the left lung constituted 0.823 ± 0.066 of that of the right lung. The mean volumes of the right and left lungs accounted for 54.9 ± 2.0 and 45.1 ± 2.0 %, respectively, of the total lung volume.

## Discussion

Proper evaluation of fetal development has relied on morphometric analysis in a reference population and constructing growth dynamics [18]. This study has presented a cross-sectional interpretation of the longitudinal growth of lung volume based on the evidence from 67 fetuses at gestations of 16–25 weeks. As a result, it is not a true representation of growth in itself, but a populational perspective. The main limitation of our study is a relatively narrow fetal age, ranging from 16 to 25 weeks of gestation. Furthermore, another partial limitation is that all measurements were conducted by a single observer in a blind fashion.

In the medical literature only Gerards et al. [6] differentiated between male and female fetuses, and did find statistically significant sex differences concerning the fetal lung volumes, which were approximately 4.3 % greater in males than females. This remains contradictory to most of the previous studies, which had reported fetal pulmonary volume to be independent of sex [5, 6, 9, 11, 19–22], and to our findings in the material under examination.

Our findings confirmed that the fetal pulmonary volume was statistically greater on the right than on the left, in keeping with all authors using both ultrasound [3, 6, 9, 12, 19, 20, 22–24] and MRI [5, 18, 25] methods.

To date, fetal lung volume has indirectly been assessed in utero using methods of three-dimensional ultrasound and MR imaging. There have been the three methods of estimating fetal lung volume using three-dimensional ultrasound: by subtraction of the heart volume from the thoracic volume [10, 21], by the multiplanar technique [6, 11, 19], and by the rotational technique with VOCAL [9, 12, 22, 23, 26]. With the use of the integrated three-dimensional ultrasound method, Lee et al. [10] and Laudy et al. [21] calculated the total fetal lung volume by subtracting the heart volume from the internal thoracic volume measured between the clavicles and diaphragm. In the material of Lee et al. [10], including 78 fetuses aged 14–40 weeks, the total lung volume varied from 2.4 to 148 ml. Of note, in the age range of 16–25 weeks, the total lung volume averaged 6.83 ml at 16, 10.61 ml at 18, 12.82 ml at 19, 15.22 ml at 20, 17.84 ml at 21, 20.67 ml at 22, 23.7 ml at 23, 26.43 ml at 24, 30,38 ml at 25, and 34.03 ml at 26 weeks. According to Laudy et al. [21], the total lung volume in 29 fetuses aged 20–38 weeks ranged between 13 and 96 ml, in accordance with the linear regression *y* = –84.83 + 4.54 × age (*R* ^2^ = 0.92; *P* < 0.001). During the second half of pregnancy (20–34 weeks) the total pulmonary volume increased approximately seven times [27]. The main criticism of this method results in overestimating the total lung volume because of many mediastinal structures, being included in the calculation. Indeed, when compared to the afore-mentioned results obtained by Lee et al. [10] and Laudy et al. [21], in the material under examination the total pulmonary volume in fetuses aged 16–25 weeks was much smaller, ranging from 2.67 ± 0.47 to 15.22 ± 5.58 cm^3^. Besides, in spite of stepping through the thorax, the heart volume, ranging widely between systole and diastole, was not corrected for fetal heart action. On the contrary to those authors [10, 21, 27], some clinicians [11, 19, 24] separately outlined and scanned the right and left pulmonary volumes, without taking into account the mediastinal volume. In that multiplanar technique, once the contour is drawn, it cannot be further modified. Furthermore, this method tends to underestimate pulmonary lung volume, because the superior and inferior anatomical limits are set at the levels of the fetal clavicle and at the diaphragmatic dome, respectively. According to Pöhls and Rempen [11], in 57 fetuses at gestations between 20 and 34 weeks, the mean lung volume varied from 5.9 to 37.2 ml on the right, and from 4.1 to 28.0 ml on the left. During that time, the total mean lung volume ranged from 9.9 to 65.3 ml. In the longitudinal observational study of Bahmaie et al. [19], including 58 fetuses aged 18–41 weeks, the mean pulmonary volume increased in a non-linear way, varying from 1.69 to 60.2 ml for the right lung, and from 1.18 to 56.26 ml for the left lung. Our results have better correlated with numerical data reported by Bahmaie et al. [19] than those presented by Pöhls and Rempen [11]. In the present study, between 16 and 25 weeks of gestation, the lung pulmonary volume rose from 1.43 ± 0.25 to 8.45 ± 2.66 cm^3^ on the right, and from 1.24 ± 0.22 to 6.78 ± 3.03 cm^3^ on the left. Of note, Gerards et al. [6] confirmed a sevenfold increase in right and left lung volumes between 20 and 34 weeks of gestation.

Kalache et al. [12] were the first to introduce the rotational method with VOCAL for three-dimensional ultrasound estimation of fetal lung volume. Both Kalache et al. [12] and Moeglin et al. [23] emphasized that both the conventional multiplanar method and the rotational technique with VOCAL produced similar results, thereby could be used interchangeably. On the other hand, due to the VOCAL technique the entire lung is visualized simultaneously with its lowermost parts that extend below the diaphragmatic dome, and after the initial volumetric calculation, an examiner can subsequently modify the lung contour, so as to obtain a much more precise final measurement [9, 22, 26]. With the use of the VOCAL technique, Ruano et al. [9] studied pulmonary volumes in 146 fetuses aged 20–37 weeks. The mean right, left and total pulmonary volumes varied from 5.37 to 46.06 ml, from 4.66 to 37.34 ml, and from 9.95 to 84.35 ml, respectively. The volumetric growth followed the exponential functions: *y* = exp [4.07/(1 + exp (21.90 − age/5.44))] for the right lung, *y* = exp [3.82/(1 + exp (22.03 − age/5.17))] for the left lung, and *y* = exp [4.72/(1 + exp (20.30 − age/6.05))] for both lungs. Peralta et al. [22] calculated pulmonary volume in 650 fetuses at the age of 12–32 weeks, in which the volumes increased as follows, from 0.6 to 6.3 ml for the right lung, from 0.6 to 4.6 ml for the left lung, and from 1.6 to 10.9 ml for both lungs. The best fit to the means of the pulmonary volume was yielded by the following regressions: *y* = 8.486164 − 1.070747 × age + 0.003578 × age^3 ^− 0.000059 × age^4^ for the right lung, *y* = 17.686614 − 2.777019 × age + 0.116851 × age^2 ^− 0.000027 × age^4^ for the left lung, and *y* = 41.822982 − 6.536788 × age + 0.273906 × age^2 ^− 0. 000069 × age^4^ for both lungs.

According to Kasprian et al. [28], MRI provides clinicians with detailed structural and unique biochemical and functional information, thereby being definitely a valuable adjunct to the diagnostic repertoire in the evaluation of lung development in the human fetus. MRI-based measurements of pulmonary lung volume are used in some centers to evaluate complex fetal thoracic abnormalities that cannot be precisely assessed with three-ultrasound alone [25]. Basing on MRI, Cannie et al. [5] evaluated pulmonary volumes in 105 fetuses at 19–40 weeks with normal lung development. Right and left lung volumes increased with age in accordance (*r* = 0.81) with the following linear fits: *y* = –43.67 + 2.42 × age for the right lung, and *y* = –32.82 + 1.82 × age for the left lung. Osada et al. [29] quantified with MRI total pulmonary lung volume in 58 fetuses at gestations of 24–39 weeks. Although the best fit for total lung volume was represented by linear regression *y* = (2.41 × age) − 37.6, its correlation with age was rather poor (*r* = 0.537). On the contrary, MRI results obtained in 215 normal fetuses aged 21–38 weeks showed the spread of pulmonary lung volumes, according to the power fit *y* = 0.0033 × age^2.86^ (*R* ^2^ = 0.58) [18]. Ward et al. [25] calculated MRI-based total fetal lung volume in 30 fetuses at gestations of 17–36 weeks. It was found to range from 2 to 110 ml with the fitted quadratic dependency *y* = 26.3 + [3.3 × (age − 25 weeks)] + [0.167 × (age − 25 weeks)^2^]. Some studies [2, 7, 30] confirmed a very strong concordance (*r* = 0.92–0.95) between three-dimensional ultrasonography and MRI in the evaluation of fetal pulmonary volume.

All the presented above growth dynamics for fetal pulmonary volumes do not resemble our completely novel quantitative patterns, computed in this study. The best fits for fetal pulmonary volumes as functions of gestational age have been three-degree polynomial (cubic) models. The volumetric growth of the right and left lungs was modeled by the following functions *y* = –1.592 + 0.0007 × age^3^ ± 0.851 (*R* ^2^ = 0.84), and *y* = –1.110 + 0.0005 × age^3^ ± 0.794 (*R* ^2^ = 0.78), respectively. Furthermore, the total pulmonary volume followed the model *y* = –2.729 + 0.0012 × age^3^ ± 1.598 (*R* ^2^ = 0.83). It should be emphasized that coefficients of determination (*R* ^2^) attained much higher values than those reported by other authors [5, 9, 18, 22, 29].

In the material under examination, we indicated the proportionate volumetric growth of the two lungs, in keeping with some authors [1, 6, 18, 22, 26, 31], who found a constant linear regression between left and right pulmonary volumes throughout pregnancy. The left-to-right lung volume ratio was stable throughout the analyzed period, and averaged 0.823 ± 0.066. As a result, the right and left lungs accounted for 54.9 ± 2.0 and 45.1 ± 2.0 %, respectively, of the total pulmonary volume. In the material of Peralta et al. [22] and Rypens et al. [18], the left-to-right lunge volume ratio attained the values of 0.74 and 0.78, respectively. According to Gerards et al. [6], the left and right lung volumes constituted 44 and 56 %, respectively, of the total lung volume.

Having discussed the quantitative growth of fetal pulmonary volume, we would like to emphasize some relevance of our findings. To the best of our knowledge, this paper is the first anatomical study to endow us with direct pulmonary volume measurements. As a consequence, our results are not affected by many disadvantages typical of three-dimensional ultrasound (shadowing of the spine, scapula and ribs; suboptimal image quality; fetal respiratory, cardiac, and body movements) or MRI (cardiac cycle, fetal respiration) [11, 20, 25]. The present nomograms (Figs. 3, 4) display lung volumes and improve our knowledge of pulmonary quantitative morphology in formalin-fixed human fetuses. The right, left and total pulmonary volumes, obtained in this study may serve as a useful reference to anatomists dealing with developmental growth patterns in the fetus. Of note, since formalin immersion little influences lung volumes in the sealed thoracic cavity [1, 16, 30], the results obtained in this study can directly be adapted in vivo to the fetus. Both prenatal ultrasound and MR imaging have been proved to enable the diagnosis or exclusion of pulmonary hypoplasia, in which our quantitative data, as relevant fetal age-specific references for pulmonary volumes may be reliably helpful. Therefore, in the MRI material of Osada et al. [29], there was a significant intergroup difference in lung volume between fetuses with good respiratory outcome (38.3 ± 1.7 ml) and fetuses with severe respiratory disturbance (16.5 ± 2.7 ml). In the latter, the best fit for total lung volume followed the linear function *y* = 0.97 × age − 14.0 (*r* = 0.378). Furthermore, some authors [5, 32, 33] evaluated by MRI lung volumes in fetuses with congenital diaphragmatic hernia. With relation to the location of congenital diaphragmatic hernia, the contralateral lung volume was considerably greater than ipsilateral lung volume.

## Conclusions

1. No sex differences are found between the lung volumes in the fetus.
2. The growth of fetal lung volume follows a three-degree polynomial function.
3. Throughout the analyzed period the two lungs grow proportionately to each other, with the volumetric predominance of the right lung.
4. The lung volumes in the fetus are of great relevance in the evaluation of the normal pulmonary growth and the diagnosis of pulmonary hypoplasia.
